# The Effectiveness of Pre- and Postoperative Infliximab in Controlling Behçet's Disease Posterior Uveitis in Patients Undergoing Vitrectomy: A Preliminary Study

**DOI:** 10.1155/2017/8168369

**Published:** 2017-04-04

**Authors:** Hossam El Din Mohamed Khalil, Heba A. El Gendy, Hala Ahmed Raafat, Hazem Effat Haroun, Tamer Atef Gheita, Hossam Mahmoud Bakir

**Affiliations:** ^1^Department of Ophthalmology, Faculty of Medicine, Beni Sweif University, Beni Sweif, Egypt; ^2^Department of Ophthalmology, Faculty of Medicine, Cairo University, Giza, Egypt; ^3^Department of Rheumatology and Rehabilitation, Faculty of Medicine, Cairo University, Giza, Egypt

## Abstract

*Purpose.* To evaluate the short-term effectiveness of infliximab in controlling ocular manifestations in Behçet's Disease (BD) patients candidate for pars plana vitrectomy, if given in a regimen before and after the planned procedure. *Patients and Methods.* 30 eyes of 27 adult male BD patients with a mean age of 35.56 yrs presented with refractory posterior uveitis not responding to immunosuppressive drugs and candidate for vitrectomy were included. Infliximab was given in a dose of 5 mg/kg intravenous infusion once every two weeks for 3 treatment sessions before the intended vitrectomy followed by 3 treatment sessions at two-week intervals, after vitrectomy. *Results.* Improvement of ocular manifestations was noted in all eyes, with complete resolution in 26 eyes (87%). Visual acuity improved from 0.23 ± 0.11 to 0.38 ± 0.17 (*p* ≤ 0.2), ESR decreased from 65.92 mm/hr ± 17.32 SD to 24.93 mm/hr ± 5.28 SD at the last treatment cycle (*p* ≤ 0.1). The mean daily dose of systemic corticosteroids was tapered from 44.54 mg/d ± 2.89 to 8.48 mg/d ± 6.38 (*p* ≤ 0.2), and no relapses were noted during the follow-up period. *Conclusion.* Infliximab may be safe and effective in controlling posterior uveitis and inducing remissions if given in a regimen before and after vitrecomy in BD patients.

## 1. Introduction

Behçet's disease (BD) is a chronic multisystem inflammatory disorder with ocular involvement in 30–70% of cases. Ocular manifestations may present as anterior uveitis and more commonly as posterior or panuveitis, whereas vasculitis remains the main pathology leading to vision-threatening complications [1–3].

Different treatment modalities have been proposed in BD in which the primary goal of treatment is to control the disease activity and to induce long-term remissions, whereas corticosteroids and other immunosuppressive agents have been proposed as the cornerstone for treatment [4].

Tumor necrosis factor (TNF-*α*) is an inflammatory cytokine that has been noted in uveitis-induced experimental animal studies, together with other cytokines, with documented increased serum and aqueous humor levels in the eyes with active posterior uveitis, whereas the blockade of these inflammatory mediators has been postulated to provide better control in BD patients by many investigators [5, 6].

Infliximab, a monoclonal antibody to TNF-*α*, is a biologic agent that has been approved for use in rheumatoid arthritis, Crohn's disease, and ankylosing spondylitis and has been proposed for the treatment of different types of ocular inflammation through binding and neutralizing the effect of TNF-*α*, with promising results [7].

According to EULAR recommendations, infliximab or cyclosporine A, in combination with azathioprine or interferon-alpha together with corticosteroids, has been recommended for the treatment of ocular Bechet's disease with severe posterior segment involvement [8].

The aim of this work is to evaluate the short-term efficacy of intravenous infliximab in controlling ocular manifestations in BD patients who are candidates for vitrectomy, if given in a regimen before and after the planned procedure.

## 2. Patients and Methods

### 2.1. Study Population

The current study protocol proceeded in accordance with the ethical standards in the Declaration of Helsinki 1964 [9].

In this prospective, nonrandomized, interventional series of cases, 30 eyes of 27 adult male BD patients, aged from 27 to 42 years (mean 35.56 ± 5.11 SD), with a disease duration ranging between 3.5 and 10.5 years (mean 7.05 ± 2.22 SD) were included in the recruitment from patients attending the rheumatology and rehabilitation and ophthalmology outpatient clinics and inpatient department in Kasr-Al-Aini and Beni-Sweif faculty hospitals, during the period from February 2014 to January 2016 (ISRCTN registry ISRCTN20489230).

All patients were candidates for pars plana vitrectomy due to their refractory posterior uveitis not responding to immunosuppressive drugs.

All patients were requested to sign an informed consent at the start of the current study regarding their acceptance of participation, the detailed surgical procedure and the protocol of medications as well as the follow-up protocol, and the possible complications.

### 2.2. Data Collection and Clinical Assessment

Full history taking, regarding the disease manifestations, disease duration, and previous immunosuppressant therapy, was implemented. Current medications received by the patients were considered, and patients receiving corticosteroids for management of their disease were not excluded. Full comprehensive clinical examination and skin pathergy test were conducted for all participants, whereas the diagnosis of BD was confirmed based upon satisfying the set of diagnostic criteria published by the International Study Group for Behçet's disease in 1990 [10].

### 2.3. Investigations

Blood specimens were collected after an overnight fast. All patients had the following laboratory investigations prior to the initiation of the first injection and prior subsequent doses to monitor drug-related side effects, that is, complete blood count, liver function tests, platelet count, erythrocyte sedimentation rate, antinuclear antibody level, renal function tests. Chest X-ray was performed in the study to exclude chest infection or TB, as well as tuberculin skin test, where patients with negative skin tests were only included.

### 2.4. Ophthalmological Examination

All patients underwent a full ophthalmologic examination, that is, best-corrected visual acuity (BCVA) testing, slit-lamp anterior segment examination, slit-lamp biomicroscopy, tonometry, and indirect ophthalmoscopy. Other investigations including fundus photography, fluorescein angiography, and ultrasonography were performed as indicated.

### 2.5. Indications of Pars Plana Vitrectomy

The eyes with refractory posterior uveitis not responding to immunosuppressive drugs, associated with dense vitreous opacities interfering with visual potentials, epiretinal membranes with associated cystoids macular edema (CME), or impending traction on the retina, were recruited thereafter for the proposed treatment protocol.

### 2.6. Exclusion Criteria

Patients were excluded when they had concurrent active infection, end-stage disease with no light perception attributable to retinal ischemia or optic atrophy, history of tuberculosis, positive tuberculin skin test, or bilateral irreversible blindness, impaired liver functions, leucopenia, and thrombocytopenia. Patients receiving immunosuppressant drugs other than corticosteroids were excluded as well.

### 2.7. Exposure

Infliximab was given in a dose of 5 mg/kg intravenous infusion over a three-hour period once every two weeks for 3 treatment sessions prior to the planned pars plana vitrectomy, after stoppage of other immunosuppressant drugs and keeping the patients on their pre-exposure daily doses of corticosteroids. All patients underwent vitrectomy operation, and vitreous opacities as well as epiretinal membranes were managed accordingly. Infliximab was then given in a dose of 5 mg/kg intravenous infusion once every two weeks for 3 treatment sessions after the surgical intervention.

Patients were observed for 1 hour after stopping infusion for adverse effects. Infusions were followed by maintenance treatment of oral prednisone that was tapered accordingly. No patients were treated with topical steroids or retrobulbar steroid injections.

### 2.8. Follow-Up

Patients were followed up for a mean period of 16.06 ± 1.96 weeks following the last treatment cycle. The primary outcome parameters were a clinical response to treatment measured as change in best-corrected visual acuity (BCVA) using a Snellen chart (on a scale of 10), signs of inflammatory activity, adverse events, reduction in concomitant corticosteroid requirements, and reduction in ESR. Unexpected complaints and complications were recorded as adverse events. The exceptions were mild flu-like illness and mild injection site erythema.

The results before the initiation of the first treatment cycle were compared with those at the end of the follow-up period. Moreover, the disease activity was defined as cells and flare in the anterior chamber, vitreous cells, retinal perivascular sheathing, retinal infiltration, new retinal hemorrhages, or optic papillitis, according to the Standardization of Uveitis Nomenclature (SUN) Working Group terminology [11].

### 2.9. Statistical Analysis

All statistical calculations and analyses were done using computer program SPSS (Statistical Package for the Social Science; SPSS Inc., Chicago, IL, USA) version 11. The data were statistically described in terms of mean ± standard deviation (± SD), median and range, or frequencies and percentages when appropriate. The Student *t*-test for paired data was used for analyzing the collected data. A difference was considered to be statistically significant (S) when the probability (*p* value) was ≤0.05.

## 3. Results

In this prospective, nonrandomized, interventional case series study, 30 eyes of 27 adult BD male patients aged from 27 to 42 years (mean 35.56 ± 5.11) and a disease duration ranged from 3.5 to 10.5 years (mean 7.05 ± 2.22), presented with refractory posterior uveitis not responding to immunosuppressive drugs, were included in the recruitment for pars plana vitrectomy procedure under a proposed treatment protocol in the form of 5 mg/kg intravenous infusion of the drug infliximab over a three-hour period once every two weeks for 3 treatment sessions prior to the planned pars plana vitrectomy, followed by postvitrectomy 3 treatment sessions once every 2 weeks.

The demographic data and clinical presentation and the preoperative medications for recruited patients are summarized in Tables 1 and 2, respectively.

Patients were followed up for a period ranging from 12 to 20 weeks (mean 16.06 ± 1.96), after the last treatment cycle for the improvement of ocular inflammatory reaction and BCVA as the primary outcomes and for the alterations in ESR levels, dose of systemic corticosteroids, and the occurrence of relapses, as well as the documentation of possible drug-related adverse reactions.

No intraoperative or postoperative complications were encountered in any of the participants with all the eyes showing a satisfactory postoperative course.

Subjective improvement in visual symptoms was reported by all patients within 24 hours after the first infusion, whereas objective improvement of ocular manifestations was detected after the planned treatment regimen in all the eyes, with complete resolution of inflammatory signs achieved in 26 eyes (87%) by the end of the follow-up period.

In 4 eyes, a failure to achieve complete resolution of inflammatory manifestations in the form of persistence of anterior chamber and vitreous cells was noted, despite the relative improvement as compared to the initial pretreatment condition.

All the eyes showed an improvement of BCVA by 1–3 lines on a Snellen chart (mean 2.23 ± 0.67), and the mean BCVA improved from 0.23 ± 0.11 to 0.38 ± 0.17 by the end of the follow-up period that was considered not statistically significant (*p* ≤ 0.2).

ESR considerably decreased from a mean pretreatment value of 65.92 mm/hr ± 17.32 SD to a mean value of 24.93 mm/hr ± 5.28 SD by the end of the last treatment cycle (*p* ≤ 0.1).

The mean daily dose of systemic corticosteroids was tapered throughout the follow-up period in all patients with stoppage of systemic corticosteroids achieved in 5 patients (18.5%) by the end of the follow-up period. The detailed pre- and posttreatment corticosteroid doses are shown in Table 3.

No eyes showed signs of reactivation, that is, relapses throughout the follow-up period, and the introduction of additional treatment modalities was not needed in any of the participants as well.

Regular monitoring of patients throughout the treatment course revealed no serious complications necessitating the discontinuation of intravenous infliximab infusion, whereas the proposed treatment protocol was conducted in all the recruited participants.

## 4. Discussion

The procedure of pars plana vitrectomy has been advocated in selected cases with intractable posterior uveitis in BD patients not responding to different treatment modalities including immunosuppressant agents and/or with the development of sight-threatening complications, that is, epiretinal membranes with tractional detachment that the persistence of vitreous components has been postulated to reactivate secondary immune response, with its high levels of inflammatory cytokines [12, 13].

The role of T lymphocytes in the pathogenesis of BD and the documentations of high serum levels of inflammatory cytokines in patients with active disease as well in aqueous samples of the eyes with activity has been postulated by many investigators, whereas tumor necrosis factor*-alpha* (TNF-*α*), an inflammatory cytokine, proved to have a role in the disease activity, which highlight the role of biologic agents, for example, infliximab as a promising treatment modality in intractable cases, through binding TNF-*α* and its soluble receptors [14, 15].

Despite being highly effective in cases with severe ocular inflammatory conditions and retinal involvement, the high cost effect of the drug infliximab might stand as a limitation in considering the drug in the treatment protocols of patients with ocular Bechet in developing countries.

Another questionable issue regarding the use of infliximab is the necessity of a maintenance treatment protocol after the initial control of the ocular condition to prevent relapses, which again raised the high cost effect problem for the use of the drug in centers with no governmental medical insurance [16, 17].

In the present study, we evaluated the effectiveness of a proposed treatment regimen in the BD eyes scheduled for vitrectomy due to intractable vitritis not responding to medical treatment, in the form of 3 preoperative treatment cycles followed by 3 postoperative maintenance cycles with 2-week intervals, in controlling ocular inflammatory response and inducing long-term remissions.

To our knowledge, this is the first study considering the evaluation of the efficacy of pre- and postvitrectomy infliximab in controlling cases with posterior uveitis resisting medical treatment in BD patients.

In the current study, all the eyes showed considerable improvement in ocular symptoms and signs of inflammation with 87% of the eyes showed complete resolution by the end of the follow-up period. Moreover, a decline in ESR values, denoting the relative control of the inflammatory cascade, was achieved in all patients.

A considerable result in our work is that the daily corticosteroid dose was tapered in all cases according to ocular and systemic conditions and was stopped in 5 patients by the end of the follow-up period, without the documentation of any activity after reducing or even discontinue the daily dose of corticosteroids.

Despite the debate regarding the safety and efficacy of vitrectomy in the eyes with active inflammation, with published reports documenting the occurrence of severe inflammations postvitrectomy, as well as the reported risk of postoperative hypotony [18–20], our results seemed to be promising with good control of the inflammatory process in all cases.

The results in the current study are still comparable with the data published by Heiligenhaus et al. [21], who recommended a preoperative control of intraocular inflammation prior to the vitrectomy procedure to decrease the risk of complications, and Mesquida et al., with reported favorable results following pars plana vitrectomy in the Bechet eyes with no reported postoperative complications and recorded control of ocular inflammatory condition and the reduce in postvitrectomy systemic treatment [22].

Our results, regarding the efficacy of infliximab in controlling ocular inflammatory conditions, improving VA, and its influence on other immune suppressants, with subsequent tapering of systemic doses of corticosteroids used by all patients after the administration of infliximab, are comparable with published data by many investigators [17, 23–26].

In the present study, no relapses were recorded throughout the follow-up period, that is, 16.09 weeks after the last treatment cycle, which is not comparable with previous published data that reported the occurrence of relapses at a reduced rate following either pars plana vitrectomy or infliximab therapy [15–17, 21].

In our opinion, the occurrence of no relapses in the present study may be attributed to both the combination of pars plana vitrectomy with subsequent removal of cytokines loaded vitreous and the initiation of preoperative infliximab therapy aiming for preoperative control of the inflammatory activity, followed by postoperative treatment cycles.

## 5. Conclusions

In our experience, infliximab may be safe and effective in controlling posterior uveitis if given in a regimen before and after vitrectomy in BD patients allowing the reduction of corticosteroids and immunosuppressive drugs required to control the disease, as well as in inducing long-term remission; however, a proper patient selection and meticulous follow-up during the treatment course should be considered to overcome the drug adverse reactions, for example, infections.

Despite being statistically not significant, however, the clinical improvement in BCVA, the decreased ESR values, the reduced corticosteroids' daily doses, and the experience of no relapses after the discontinuation of the drug may encourage the proposed treatment regimen in cases candidate for surgical intervention with possible promising results.

Further studies regarding this proposed treatment regimen on a larger number of patients as well as longer follow-up periods are recommended for further evaluation of the long-term results particularly its influence on the rate of relapses and the necessity of maintenance doses of infliximab to stabilize the disease condition.

## Figures and Tables

**Table 1:** Demographic data and clinical presentations.

Demographic data	Mean ± SD	Range
Age (years)	35.56 ± 5.11	27–42
Duration (years)	7.05 ± 2.22	3.5–10.5

Clinical presentations	*N* ^∗^	%

Ocular manifestations	27	100%
Anterior uveitis	5	18.5%
Posterior uveitis	27	100%
Vitreous cells & opacities	27	100%
Retinal vasculitis	5	18.5%
Epiretinal membranes	8	29.6%
CME	2	7.4%
Extraocular manifestations	15	55.6%

^∗^Number of patients.

**Table 2:** The average daily doses of preoperative medications.

Medications	*N*	%	Dose^∗^
Corticosteroids	27	100%	44.54 ± 2.89
Cyclosporin A	7	26%	200 ± 33.66
Colchicine	20	74%	0.93 ± 0.3
Azathioprine	5	18.5%	139.4 ± 19.5
Cyclophosmaide	5	18.5%	719 ± 48.01

^∗^mg/day.

**Table 3:** Daily dose of corticosteroids, before and after infliximab treatment in the BD patients.

Daily dose	^∗^Before infliximab	After infliximab	*p* value
Min	36.02	0	
Max	48.5	20	
Mean	44.54 ± 2.89	8.48 ± 6.38	≤0.2

^∗^mg/day.
